# Effects of memory on the shapes of simple outbreak trees

**DOI:** 10.1038/srep21159

**Published:** 2016-02-18

**Authors:** Giacomo Plazzotta, Christopher Kwan, Michael Boyd, Caroline Colijn

**Affiliations:** 1 Department of Mathematics, Imperial College London, London, UK; 2 Department of Electrical and Electronic Engineering, Imperial College London, London, UK; 3 Department of Mathematics, University of Cambridge, Cambridge, UK

## Abstract

Genomic tools, including phylogenetic trees derived from sequence data, are increasingly used to understand outbreaks of infectious diseases. One challenge is to link phylogenetic trees to patterns of transmission. Particularly in bacteria that cause chronic infections, this inference is affected by variable infectious periods and infectivity over time. It is known that non-exponential infectious periods can have substantial effects on pathogens’ transmission dynamics. Here we ask how this non-Markovian nature of an outbreak process affects the branching trees describing that process, with particular focus on tree shapes. We simulate Crump-Mode-Jagers branching processes and compare different patterns of infectivity over time. We find that memory (non-Markovian-ness) in the process can have a pronounced effect on the shapes of the outbreak’s branching pattern. However, memory also has a pronounced effect on the sizes of the trees, even when the duration of the simulation is fixed. When the sizes of the trees are constrained to a constant value, memory in our processes has little direct effect on tree shapes, but can bias inference of the birth rate from trees. We compare simulated branching trees to phylogenetic trees from an outbreak of tuberculosis in Canada, and discuss the relevance of memory to this dataset.

Understanding outbreaks of an infectious disease is important for understanding how a pathogen spreads, and in determining the best steps to take to control it. Recently, the advent of next-generation sequencing has permitted the use of genomic data to assist in understanding outbreaks. Even small amounts of genetic variation within an outbreak can potentially be detected with whole-genome sequencing, and used to aid in reconstructing who infected whom^1^,^2^,^3^,^4^,^5^,^6^,^7^,^8^. Genomic data are typically analysed by inferring phylogenetic trees (phylogenies), namely trees in which the tips correspond to pathogen isolates from infected cases, and the internal nodes correspond to inferred ancestors. Phylogenies are usually rooted, binary trees. An ongoing challenge in epidemiology is to make the best use of genomic data, usually with the help of inference and analysis of phylogenetic trees that carry information on parameters including the basic reproduction number (*R*_0_)^9^,^10^.

Phylogenetic trees are related to branching trees. In a branching process without multifurcations (ie where there is a positive time interval between successive branching events), the process defines a bifurcating tree moving forward in time; internal branch lengths reflect times between infection events, and pendant branch lengths reflect the time between an infection event and a sampling event. Under good conditions (where evolution is clock-like, within-host diversity is low and where sufficient diversity accrues across sampled individuals), a timed phylogenetic tree can be seen as an approximate representation of the true branching tree, though it does not include the information of who infected whom in a direct way. Indeed, the link between pairwise genetic diversity and who infected whom has been widely studied and discussed^6^,^7^,^8^,^11^,^12^,^13^,^14^,^15^,^16^,^17^. These assumptions may break down for various reasons, but the study of branching trees remains a central tool for modelling phylogenetic trees.

Both the theory of branching processes and Kingman’s coalescent theory provide models for branching trees; these have been used to good effect in theoretical epidemiology^11^,^14^,^15^,^18^,^19^,^20^,^21^. The constant rate birth-death and coalescent processes share the simplifying assumption that distribution of times between branching events is exponential, due to the constant rates, mirroring the exponential time distribution in the susceptible-infectious-recovered (SIR)-type epidemic models that have been widely used to model the spread of infection^22^. However, exponentially distributed (memory-less) infectious periods are not very realistic for many infections^23^,^24^,^25^,^26^,^27^,^28^. Non-exponential distributions in models of the spread of infection have been a topic of study for decades, and it is well established that incorporating memory in these processes can have large effects on the models’ dynamics^24^,^26^,^29^,^30^,^31^,^32^. Non-exponential distributions, particularly in the infectious period, can also affect the estimation of *R*_0_ and other parameters^33^,^34^,^35^. The growing fields of phylodynamics and genomic epidemiology, however, have primarily used the constant rate assumption because of its tractability and the inherent additional complexities of estimation from sequence data. Recently there has been growing interest in non-Markovian processes in this context, particularly non-constant removal rates^36^,^37^,^38^. Using multiple compartments, the epidemiological coalescent can account for non-exponential durations of infectiousness and variable infectivity^15^,^19^,^21^ but in models with many compartments, the necessary inference becomes challenging due to large numbers of latent variables^15^.

Chronic bacterial infections such as tuberculosis have long and variable durations of infection. This can include a non-infectious latent period, as is the case for tuberculosis, and can also include infectiousness worsening over time. Furthermore, cases may not present clinically in the chronological order in which they were infected. An individual may be undiagnosed and infectious for months, and some infections may remain latent for variable time periods in infected individuals. Accordingly, the spreading processes of these complex infections are likely to depart substantially from the constant rate assumption, and reconstructing transmission events using the timing of case presentation is not always feasible.

Models have so far focused primarily on the branching times in phylogenetic trees, as these are natural quantities in branching processes and coalescent theory. However, it has been observed for species phylogenies that the tree shapes arising from the Yule or constant-rate birth-death processes do not fit trees from data particularly well^39^,^40^,^41^,^42^,^43^. Several studies have suggested that tree shapes carry relevant information for epidemiology^21^,^44^,^45^,^46^,^47^, and tree shapes have recently been shown to have applications to inference from phylogenies derived from both viral and macroevolutionary data^21^,^48^. Frost and Volz^21^ noted that coalescent times are not sufficient to estimate epidemiological dynamics in complex models (such as structured populations), though they do very well in simple populations. They found that high transmission in the acute stage of HIV infection affected the asymmetry and the numbers of cherry configurations in phylogenies^21^. Recently, Hagen *et al.*^48^ also found that variable speciation rates in macroevolutionary processes affect tree imbalance and produce trees that match the shapes of thousands of macroevolutionary trees better than trees from homogeneous processes. However, while asymmetry and cherry patterns capture aspects of tree shape, they do not describe it entirely. Incorporating tree shapes into the growing field of phylodynamics is an open challenge.

Here, we simulate and compare outbreaks using infectiousness functions that vary sharply over time. We compare a range of shape features of the resulting trees to each other, to trees from constant rate processes, and to trees from a tuberculosis dataset for which memory is likely relevant. We allow substantial delays between infection and infectiousness, so that the times are not exponentially distributed. This introduces memory into the process. We control either *R*_0_ (the mean number of secondary infections) or the Malthusian parameter *M* (the mean rate of growth of the process), but vary the timing of infections, and explore how this affects the shapes of the outbreaks’ branching trees. We find that contrary to our expectations, memory in the process has very little direct effect on the shapes of branching trees. Rather, it strongly affects the number of infected individuals (tips in the tree), and affects tree shapes as a consequence. It also affects estimates of the birth rate derived from branching times, and it affects the comparison between the branch timing and that expected under a Yule process. We find that phylogenies derived from data do not match the shapes of the constant-rate birth-death models even when the birth and death rates were fit to data, and we discuss whether this match should be expected.

## Methods

### Formulation and notation

We use the Crump-Mode-Jagers generalised branching process. We follow Jagers’ setting^49^: each individual is assigned a random variable *λ* modelling its life/recovery time and a point process *ξ* modelling the number of individuals that he infects and the respective infection times. The pairs (*λ*, *ξ*) assigned to different individuals are independent and identically distributed. This implies that in the process *z*(*t*), defined as the number of individuals alive at time *t*, is indeed a branching process.

In our model, *λ* is an exponential random variable representing the life span of an individual, its expectation is 1/*δ* where *δ* is the death rate. If *δ* = 0 we have trees with no death, this results in ultrametric trees, i.e. trees where the distance from an ancestor to any of the tips is the same. This removes the risk that sampling through time will bias the shape features^21^. The point process *ξ* (the new infections caused by each individual) is a non-homogeneous Poisson process with intensity *I*(*t*). The mean number of secondary infections caused by any individual (*R*_0_) is given by 
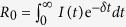
, where *t* is the time since an the individual became infected. The Malthusian parameter *M* is the finite positive solution to the equation 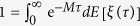 which in our setting reduces to 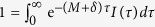. The Malthusian parameter *M* exists if *E*[*ξ*(0)] < 1 < *E*[*ξ*(∞)] < ∞, which are reasonable properties in the context of transmission trees (and we assume them throughout). The Malthusian parameter captures the growth of the process because the expectation *E*[e^−*Mt*^*z*(*t*)] converges to a constant as *t* → ∞^50^.

To convert the branching process simulation to a rooted binary transmission tree with branch lengths in units of time, we begin at the source case, adding a node each time there is an infection event. One of the descending lineages from that node corresponds to the infector (say A), and the other to the infectee (say B). The length of the branch is either (1) the time between A’s infection and A infecting B (if B is the first case A infected), or (2) the time between A’s infecting the individual she infected just prior to B and the time A infects B. The pendant branch length to the tip labelled B is the time between either (1) B’s infection and B’s sampling (if B did not infect anyone) or (2) the time between B’s last infection of someone else and B’s sampling. In this way, the branching process defines a rooted, timed, bifurcating tree. While the focus of this paper is on how the shapes of these trees are affected by the intensity function describing when infection events occur, the motivation of the work makes the implicit assumption that these shapes are made relevant because under good conditions, timed phylogenetic trees are a reasonable approximation to these bifurcating timed transmission trees.

### Simulations

Simulating stochastic branching trees under processes with memory is challenging. Gillespie-type methods do not fit the problem naturally, and steps must be taken to ensure that all events that can happen before the final simulation time have the appropriate probability of happening (affecting the conditions under which the process can be stopped). This can be very computationally time-consuming due to the variability of the tree size in branching processes. This complexity is part of the motivation for using simple intensity functions. To simulate non homogeneous trees, we wrote an iterative function that takes in input the ancestor, the start and final time of the tree, the intensity function and the life-span distribution: SimNHTree(ancestor, startTime, finalTime, int, lambda). The branching property states that the subtree generated by each daughter of the ancestor is equal, in distribution, to the whole tree in every aspect but the starting time. To use this property we first find the number of daughters of the ancestor and their birth times, simulating a non homogeneous Poisson process with intensity int. Then, for each of the ancestor’s daughters, the function SimNHTree calls itself with the daughter and its birth time as new input: SimNHTree(daughter_i_, birthTime(daughter_i_), finalTime, int, lambda). This generates the ancestor’s daughters subtrees which can be merged because we track each subtree’s ancestor and start time.

**Figure 1:**
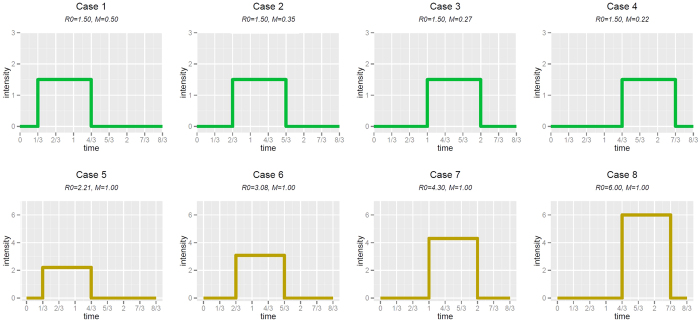
Intensity functions used in Scenario 1 and 2.

**Figure 2:**
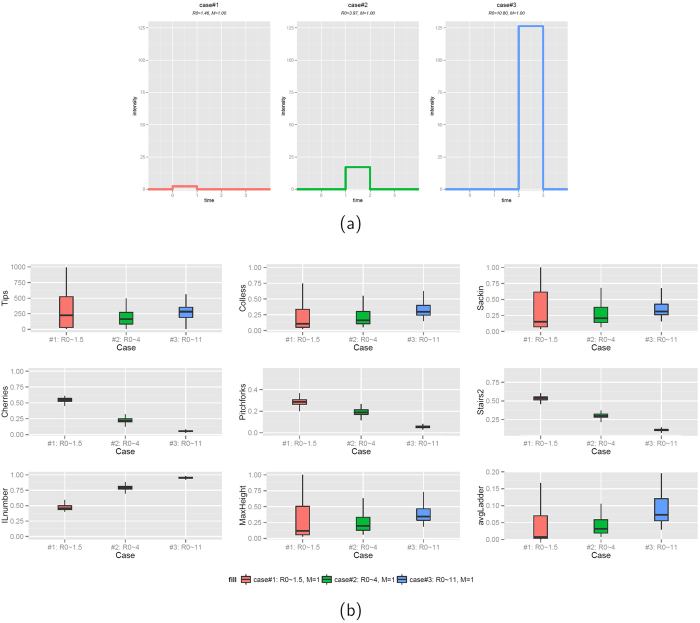
Intensity functions (a) and shapes of trees (b) from Scenario 3, in which we explore a more extreme example of memory with a high *R*_0_, high *M*, and delayed but high intensity function. (See Table 1 for parameters).

We use two different approaches to setting the stopping time: in **Scenario 1** we fix the time for each intensity function so that when *R*_0_ is the same, so is the time, and when *M* is the same, so is the time. In **Scenario 2** we tune the time to obtain, on average, trees with the desired number of tips. To do this, we use a simple algorithm that simulates a group of trees and if the total average of the tips is too high/low then the algorithm decreases/increases the final time and starts a new simulation. It stops once the average number of tips of each group is between 32 and 34 (to be comparable to our dataset) or between 98 and 102, in order to compare results in larger trees (Scenario 2-large). To exclude meaningless cases, we rejected trees with fewer than 5 tips. In each scenario, we vary the delay between becoming infected and infecting others, using different intensity functions (each labelled with a case number illustrated in Figs. 1 and 2(a)). In **Scenario 3** we increase both the delay between becoming infected and infecting others (ie location of the intensity function) and the height of the intensity function. This results in the basic reproduction number ranging more widely than in Scenarios 1 and 2. Here we also use a positive death rate, so trees are not ultrametric.

The parameters were chosen empirically, in order to explore and compare trees originated by different intensity functions, but sharing biologically-relevant measures such as *R*_0_, *M* or the size. The parameter choice was not intended to fit a specific outbreak. However, *R*_0_ values for most common pathogens including tuberculosis (*R*_0_ = 1–1.5) are in the range 1–6, with some viruses having much higher values (measles for example at *R*_0_ ≈ 20). Our choices of *R*_0_ mirror these values. The sizes of our outbreaks mirror the dataset we have, and in order that the results not be restricted to processes of this small size we also explore larger trees. Table 1 and Figs 1 and 2(a) give details of the parameters in each simulation. We compare these sets of simulations with two sets of constant-rate birth-death trees, one with with parameters matching the *R*_0_ of the trees in Scenario 1, and one with parameters estimated from our TB data using BEAST. For each case in Scenario 1, 2 and 3 we simulate 200 trees and for the two homogeneous cases we simulate 1000 trees.

The number of secondary infections per infectious case has a mean of *R*_0_ but of course it can be distributed in various ways. In the terminology of branching processes this distribution is called the offspring distribution; the constant-rate birth-death process has a geometric offspring distribution (a convolution of a Poisson number during their lifespan and an exponential lifespan). However our non-homogeneous Poisson processes, in which cases survive their infectious period, have a Poisson offspring distribution. To explore possible effects of this difference, we simulate a variant of our process in which the intensity functions varied as in Scenario 1, but we draw the numbers of secondary infections from a geometric distribution (see Supplement).

### Intensity functions: the time between infection and infecting others

For scenario 1, 2, and 2-large we choose intensity functions such that we can introduce memory while fixing *R*_0_ (cases 1–4 in Table 1), or fixing the Malthusian parameter (cases 5–8 in Table 1). It is not possible to fix both simultaneously while varying the intensity function independently. We vary the timing of infectiousness, from beginning immediately (cases 1 and 5 in Table 1) to beginning relatively late after a case was infected (cases 4 and 8 in Table 1); Fig. 1 illustrates the intensity functions for each case.

For a general step-like intensity function, *R*_0_ is given by


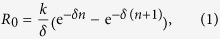


where *k* is the height of the step, *n* defines the step interval [*n*, *n* + 1] and *δ* is the death rate. The Malthusian parameter *M* cannot be written in closed form and is the solution of the following equation:


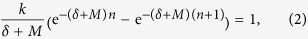


where *k*, *n* and *δ* are same as in Equation (1). Given a fixed value of *R*_0_, Eq. (1) can be used to obtain different intensity functions with the same *R*_0_, varying the parameters *n* and *k*. In a similar way if *M* is fixed, from Eq. (2) one can derive the height *k* for different values of *n*, thus defining different intensity functions with the same Malthusian parameter. In this way we derive the intensity functions in Fig. 1.

### Shape features

**Figure 3:**
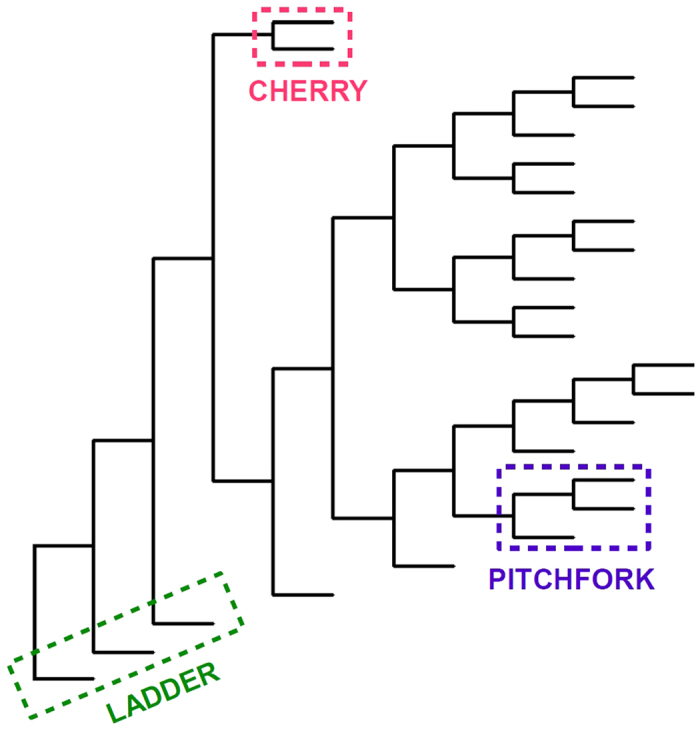
Graphical illustration of a cherry, a pitchfork and a ladder.

Many of the functions used in this paper have been collected into an R package called phyloTop. Its aim is to allow the calculation of topological properties of phylogenetic trees. It does this by allowing the calculation of certain basic properties. Three important examples of topological properties of the nodes of a tree are the number of descendants of each node (this generalises the concept of cherries), the imbalance in the number of descendants and the length of the ladder starting from that node. For a graphical representation of cherry, pitchfork and ladder we refer to Fig. 3. The package includes tools to calculate these and many others. Once these basic properties have been found it is easy to calculate whatever else may be needed. This approach is quite flexible in calculating other topological properties. phyloTop implements this practice for some common examples such as the Colless and Sackin imbalance. phyloTop is based on the R package phylobase.

We use the normalized Colless imbalance^51^,^52^, Sackin imbalance^16^,^53^, the number of cherries^21^,^54^, the number of pitchforks, a “stairness” property (stairs2), the number of internal nodes with a single tip descendent (ILnumber), and an average “ladder length” (avgLadder). A cherry is two tips with a common ancestor. A pitchfork is a configuration of 3 tips: one cherry and an additional tip with a common ancestor. They can be counted in phyloTop with nConfig (tree,3). The stairs feature is the second “stair-ness” shape defined by Norstrom^55^, namely the average of 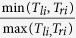 over the internal nodes of the tree. Here, *T*_*ri*_ and *T*_*li*_ are the number of tips descending from the left and right sides at internal node *i*. We define a *ladder* to be a series of of connected internal nodes, each with a single leaf descendant. The *avgLadder* is the average length of ladders in the tree.

These can all be computed in a straightforward manner in the phyloTop package. The relevant phyloTop functions were then used with a function called *treeListSummary*. As inputs, this takes a list of functions (each of which return a topological property of a tree) and a list of trees. It then returns a data frame displaying the results of applying the input functions to the input trees. phyloTop has been made available on CRAN with a standard open source licence.

We normalise the shape features by comparing them to the maximum possible value in a tree of the given size. Normalization is performed by division by the maximum possible value, which is a function of the number of tips, *n*. While the expected value of any of the shape patterns will vary with the model under which the expectation is taken, and these averages are in general challenging to determine, the maximum possible value in a tree of size *n* is straightforward for all of these shape features. Normalization is as follows: Colless (already normalized); Sackin (normalized dividing by: 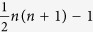); cherries (normalized dividing by *n*/2); pitchforks (normalized dividing by: *n*/3); Stairs2 (already normalized); ILnumber (normalized by dividing by *n* − 2); max height (normalised by dividing by *n* − 1), average ladder (normalized by dividing by *n* − 2).

A linear regression was performed for normalised shape feature versus the start of the intensity burst *n*. A t-test on the slope was used to infer whether the feature increased or decreased with *n*. The test and the relative p-vaue, i.e. the probabiility given the simulations that the shape feature considered is are neither increasing nor decreasing, was found with the function t.test in R^56^. In addition, in the supplement the Spearman’s correlation between shape features was computed for each scenario and case.

### Data

We use data previously described in^8^ and^47^ (Outbreak A) Briefly, the outbreak included 33 *M. tuberculosis* isolates collected in British Columbia between 2006 and 2011. Isolates were sequenced using paired-end 75bp reads on the Illumina HiSeq. The outbreak, sequences and SNPs are presented in^8^. Reads were aligned against the reference genome M. tuberculosis CDC1551 (NC002755) using Burrows-Wheeler Aligner (BWA)^57^. Single nucleotide variants were identified using samtools mpileup^58^ and were filtered to remove any variant positions within 250bp of each other and any positions for which at least one isolate did not have a genotype quality score of 222. The remaining variants were manually reviewed for accuracy and were used to construct phylogenetic trees with BEAST^59^,^60^ and MrBayes^61^. BEAST was run with the tip dates, and with birth-death serial sampling model^62^, an uncorrelated relaxed molecular clock with an exponential (1) prior on the rate, and a GTR substitution model. The MCMC chain length was 10000000 with every 1000th stored. MrBayes was run with the following options: lset Ploidy = Haploid; prset Brlenspr = clock:uniform; prset Treeagepr = Gamma(7.5, 1); prset nodeagepr = calibrated and tip dates included.

## Results

A linear model was fitted to the group of simulations cases 1–4 and cases 5–8 in order to investigate how memory, in terms of different intensity functions, may affect processes with either same basic reproduction number or same Malthusian parameters. The result of the statistical analysis is shown in Table 2.

**Figure 4:**
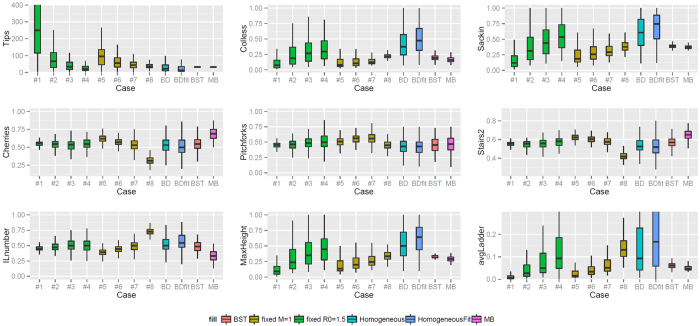
Boxplots describing shape features of branching trees from Scenario 1. Cases 1–4 have the same *R*_0_ (1.5) and cases 5–8 have the same Malthusian parameter (1). BD refers to the constant-rate birth-death process with *R*_0_ = 1.5, and BDFIT refers to the constant-rate birth-death process with parameters inferred from data. BST and MB are trees inferred from data using BEAST and MB respectively. (see Table 1)

**Figure 5:**
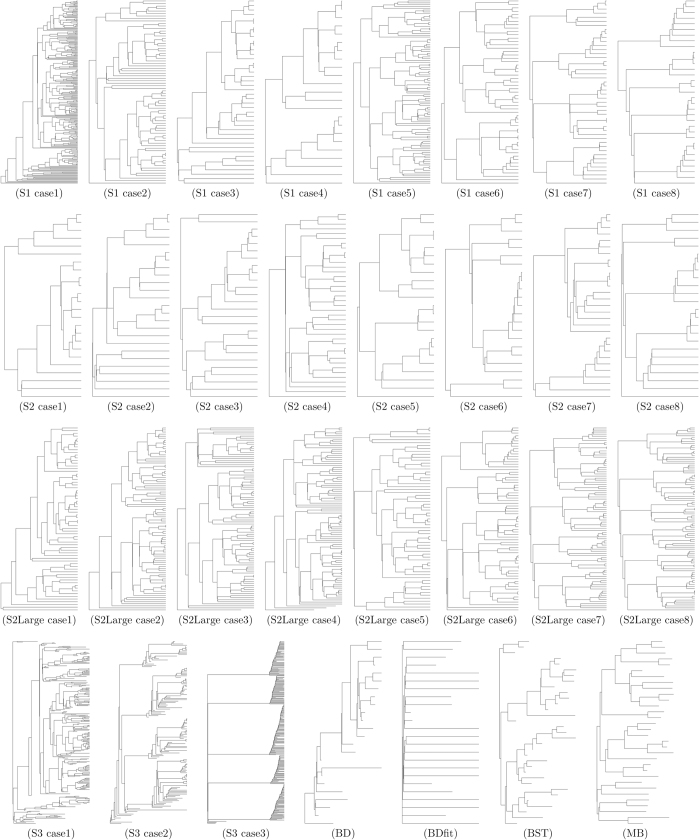
Example of trees from each scenario and case. The example trees were chosen because they have a number of tips close to the median of each group. Please note that the time (horizontal axis) does not have the same scale for different graphs.

From the simulations in Scenario 1, summarised in Fig. 4, we find that memory can affect many of the shape features we compared. In particular, as the start of the infectious period *n* moves further from the time of infection, the tree imbalance increases. A negative or null slope of both standardised Colless and Sackin imbalance is rejected with a p-value *p* < 10^−8^ for both cases 1–4 and cases 5–8. The frequency of cherries is unaffected by memory if the *R*_0_ is kept constant (*p* = 0.48). For cases 5–8 the frequency of cherries is decreasing, having rejected the hypothesis of a null or negative slope (*p* < 10^−15^). The frequency of pitchforks shows a slight increase for cases 1–4 in (*p* = 1.3 ⋅ 10^−6^) and decrease for cases 5–8 (*p* = 1.5 ⋅ 10^−4^). Similarly the “stairs 2” feature^55^ increases as *n* increases for cases 1–4 where the *R*_0_ is kept constant (*p* = 1.4 ⋅ 10^−4^) and decreases for cases 5–8 where the Malthusian parameter is constant (*p* < 10^−15^). The normalised number of internal nodes with a single tip descendant (“ILnumber”), maximum heights and average ladder length increase when both the *R*_0_ is constant (*p* 1.9 ⋅ 10^−5^, <10^−15^, 9.9 ⋅ 10^−9^ respectively) and when the Matlhusian parameter is constant (all *p* <10^−15^). Whereas it is difficult to visualise most topological differences, the increase in imbalance, standardised maximum height and average ladder can be appreciated from the example trees related to Scenario 1 in Fig. 5. for instance the tree in (S1 case5) is more balance and has smaller ladders, in proportion to its tips, than (S1 case7) or (S1 case8).

**Figure 6:**
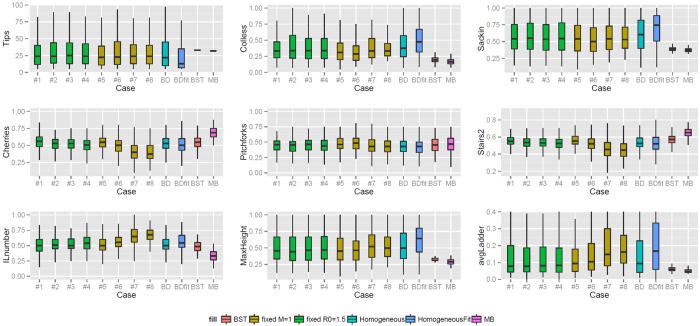
Boxplots describing shape features of branching trees from Scenario 2, where the size of the tree is controlled by varying the times of the simulations. Cases 1–4 have the same *R*_0_ (1.5) and cases 5–8 have the same Malthusian parameter (1). BD refers to the constant-rate birth-death process with *R*_0_ = 1.5, and BDFIT refers to the constant-rate birth-death process with parameters inferred from data. BST and MB are trees inferred from data using BEAST and MB respectively. (see Table 1).

However, the most dramatic difference between the various cases in Fig. 4 is in the number of tips. This was against expectation because the combinations final time-*R*_0_ and final time-*M* were kept the same in cases 1–4 and 5–8 respectively (see Table 1). Particularly in cases 5–8 which have the same Malthusian parameter, the net growth is the same up to a (usually unknown) constant, and here it seems that this constant is highly dependent on the specific intensity function. This led us to ask whether the impact of memory on tree shapes in this context is just a matter of the impact of memory on the number of tips. To explore this question, we adjusted the time periods of the simulations to allow the different cases to produce branching trees of comparable sizes (Scenario 2). Fig. 6 shows the result. We now see lower differences between the processes. In the simulations where *R*_0_ is kept constant, only the frequency of cherries, the “stairs 2” and the ILnumber show a statistically significant (*p* < 0.01) pattern. The cherry frequency and the “stairs 2” decrease (*p* of 0.2 ⋅ 10^−3^ and 0.6 ⋅ 10^−3^ respectively), whether the ILnumber increases as *n* increases (*p* = 0.3 ⋅ 10^−2^). In the cases with constant *M*, more shape features showed statistically significant patterns. As the time between infection and the start of the infectious period grows, the cherry and pitchfork frequencies together with the “stairs 2” measure decrease (*p* of 10^−15^, 0.5 ⋅ 10^−3^, <10^−15^ respectively), whether the ILnumber, the maximum height and the average ladder increase (*p* of <10^−15^, 0.3 ⋅ 10^−2^ and 0.1 ⋅ 10^−3^ respectively). Comparing to Scenario 1, adjusting for the size of the branching trees eradicates the some effects of memory, particularly in the simulations where *R*_0_ is kept constant. In Fig. 5, second row, some example trees for Scenario 2 were chosen. The shape difference between each other is not as evident as the trees from Scenario 1; there is a clear increase in the proportion of internal nodes with a one tip descendant (standardised ILnumber) from case 5 (11 nodes, 23 tips), to case 8 (14 nodes, 24 tips).

**Figure 7:**
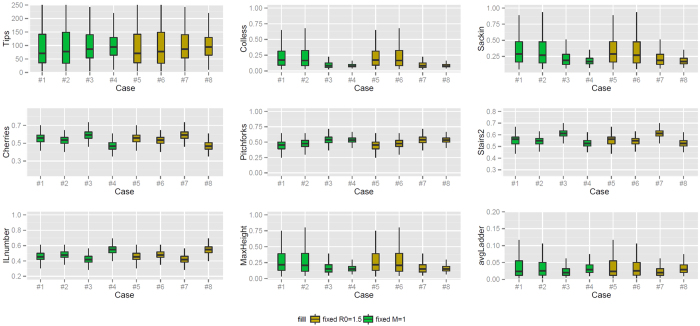
Boxplots describing shape features of branching trees from Scenario 2-large, where the tree has a mean number of tips between 98 and 102, obtained varying the simulation time. Cases 1–4 have the same *R*_0_ (1.5) and cases 5–8 have the same Malthusian parameter (1).

As in Scenario 2, Scenario2-large shows an increased level of uncertainty (high *p*); see Fig. 7 and Table 2 for a summary of the results. Compared to Scenario 2, in Scenario 2-large the Colless and Sackin imbalance for cases 5–8 is decreasing (*p* of 3.5 ⋅ 1−^−4^ and 6.0 ⋅ 10^−4^) instead of uncertain; the frequency of pitchfork for cases 1–4 increases (*p* = 7.7 ⋅ 10^−4^) and is unchanged in cases 5–8; the standardised maximum height has an opposite behaviour for cases 5–8; and the standardised average ladder for cases 5–8 does not show a statistically significant linear increase.

We used two posterior collections of phylogenetic trees derived from the Kelowna TB outbreak in Canada (see Methods). From the collection of BEAST^60^ estimates of the trees, birth and death rates were estimated. The estimated values were used to simulate the homogeneous trees in scenario BDFit, please refer to Table 1 for the input values used. Data-derived trees had slightly lower imbalance and slightly lower normalised maximum height than the simulated trees once size was controlled (Fig. 6); the two inference methods differed more with each other in several shape parameters (cherries, IL number and stairs) than the data trees differed from the simulations. Comparing to Scenario 1, the variability in the data-derived trees was typically much lower than that in the constant-rate models and more closely matched the tightly defined simulations in cases 3, 4, 7, and 8, but then the number of tips in the data is fixed, and the numbers of tips in cases 3, 4, 7 and 8 were the most narrowly distributed as well. Comparing to Scenario 2, where we controlled the average size, all of the tree shape features from the data are consistent with the simulations except for imbalance, maximum height and avgLadder feature. The BEAST trees were always closer to the constant-rate birth death model than the MrBayes trees, consistent with our having used the birth-death prior in BEAST.

**Figure 8:**
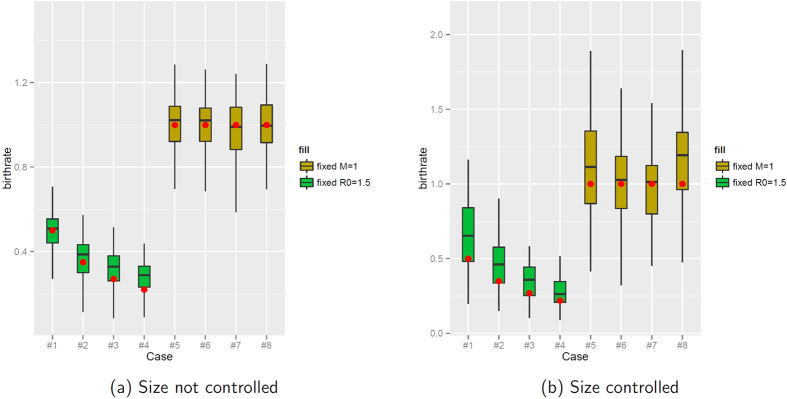
Pure birth rate fits to ultrametric simulated branching trees from Scenarios 1 (a) and 2 (b). Red dots are the correct best estimate, namely the Malthusian parameter *M* (see Table 1).

The fact that the number of tips varies dramatically while *R*_0_ or *M* are fixed means that memory affects the number of lineages in the tree. This led us to wonder whether memory would affect the results of inference approaches that assume a memory-less model and use the timing of branching events. We estimated the birth rate using the pureBirth function in the laser package in R^63^. We found that the estimates varied, and that memory resulted in some bias. Since the trees are ultrametric, the estimate of the birth rate should be equal to the Malthusian parameter. With reference to Fig. 8, the median estimate (shown by the horizontal bar in the boxplots) is too high in cases 1–4, correct in cases 5–8 in Scenario 1, but too high in cases 5 and 8 in Scenario 2. We also used Pybus’ *γ*^64^ to analyse the timing of branching events; this is possible for the ultrametric trees (Scenarios 1 and 2). We found that both memory, as we have explored it, and the distribution of the number of secondary infections, can affect whether trees appear consistent with the Yule model (see Supplementary Material).

We compared branching trees from a set of more extremely varying intensity functions such that the *R*_0_ values differed greatly. Fig. 2(a) illustrates the intensity functions, which range from infectivity beginning immediately to infectivity beginning much later. We found that high *R*_0_ values combined with a late intensity function resulted in marked differences in tree shapes, particularly in the numbers of cherries, pitchforks, the stairs feature, and the IL number, all with *p* < 10^−15^. The marked difference in shape is also evident in the examples in Fig. 5. Imbalance is increasing with *R*_0_, with *p* 8,06 ⋅ 10^−10^ and 4.1 ⋅ 10^−3^ for Colless and Sackin respectively, as well as the maximum height (*p* = 2.0 ⋅ 10^−9^). The number of tips and average ladder length are the only two shape features with no statistically significant difference among the three cases (*p* 0.87 and 0.06 respectively). A long delay between infection and start of infectious period causes the tree have only a few individuals with numerous offspring as in (S3 case2) and (S3 case3), compared to a more “normal” shape of (S3 case1). The extreme case (S3 case3) is composed by a handful of long caterpillars which imply low frequency of cherries and pitchforks and a high ILnumber, validating the results in Fig. 2.

Some of the shape features are naturally related to each other. For instance, connected “ladder” configurations will occur more frequently in imbalanced trees and cherries will be more numerous in balanced ones. Among measures of balance, Rogers^65^ showed high correlation between Sackin and Colless imbalance, under the equal rates Markov model and the equal probability model. With a simulation approach, Shao and Sokal^66^ evaluated the correlation matrix of nine indices of tree balance under the equal probability model. Similarly, they found that Colless and Sackin are highly correlated with each other.

We explored these correlations across our simulations and data (Supplementary Figures S3–S6). We found that the correlations are remarkably preserved across the simulations, but that Case 8 and the latter 2 cases from Scenario 3 (all with high *R*_0_ and delayed transmission), as well as the data, had correlation patterns which differed from the rest. Colless and Sackin imbalances are highly correlated in every simulation case, scoring a minimum of 0.88 (*p* < 10^−15^) in Scenario 2 case 8. We did not find negative correlations between cherries and imbalance, though cherries indicate symmetry (near the tips) and imbalance indicates asymmetry (over the whole tree). Unlike other cases, case 8 consistently has a negative correlation between the ILnumber feature and the pitchforks: −0.82 (*p* < 10^−15^) in Scenario 1 and −0.52 (*p* = 3, 5 ⋅ 10^−12^) in Scenario 2. This negative correlation also occurs in the high *R*_0_ cases from Scenario 3. In both data-derived groups of trees, the ladder numbers were not correlated with the imbalance measures whereas in most simulations these were tightly correlated. In the data-derived trees there were significant negative correlations between the stairs feature and the imbalance, height and ladder number, which were not present in any of the simulations. The data-derived trees had a weaker correlation between the ladder number and the imbalance than the simulations. In Scenario 3, many small but consistent correlations amongst tree shapes are reversed compared to the rest of the simulations; in particular, imbalance and tree height are not negatively correlated with cherries, pitchforks and stairs. These trees can have a high imbalance simultaneously with high values of symmetric shapes such as cherries, for example. There are strong negative correlations between the number of ladder nodes (ILnumber) and the cherries and pitchfork numbers.

Overall, when we compare how shape features have depended, or not depended, on the variations we have explored, we note that there are several shapes whose distributions were quite tightly constrained by data compared to their variability in simulations. These were the maximum height of the tree, the avgLadder feature and both measures of imbalance. In contrast, several shape features emerge as being sharply determined by the combination of high *R*_0_ and *M*: the numbers of cherries and pitchforks, the stairs feature, and the number of internal nodes with a single tip descendant (ILnumber).

## Discussion

A “process with memory” is simply a process that is non-Markovian, and there are many ways that memory can be introduced. Here, introducing memory in a simple way affected many shape features of branching trees, but also affected the timing of new infections (via the intensity function), the offspring distribution, the Malthusian parameter and the tree size distribution. This makes disentangling the effects of non-exponential waiting times on tree shapes quite complex. We have attempted to construct our study to best explore these different effects, and within this context we have found that tree shapes are quite robust to the non-exponential waiting times we have used. However, the memory in our processes did affect estimates of the birth rates and the Pybus *γ* statistic.

We explored memory using a collection of simple step-like intensity functions with a delay between a host becoming infected, and infecting others. When the delay is much larger than the pulse width (the duration of infectiousness), the branching events of each individual occur in a short time compared to the individual’s life span, very much like a burst. In the limit where the “burst” becomes very short, each individual spawns offspring at a fixed time after infection. In this case, when we observe the tree, each individual has either reached this time, infected others and completed the infectious burst or not. In such a tree, shape features such as imbalance, the cherry-to-tips ratio and so on remain essentially unchanged over time. In contrast, if the delay is comparable to the pulse width, the first offspring of an individual may infect others before her “youngest sister” is born. This overlap can influence the shape because some part of the tree may not be born when the tree is observed at the cut-off time or when a chosen number of individuals is reached.

Like most other works in the field of phylodynamics, we have implicitly assumed that the true branching tree, or at least a good posterior distribution approximating it, can be estimated using pathogen genetic data. This is a limitation, as branching events in phylogenies may not always correspond to transmission events in the outbreak. However, when within-host diversity is low, the pathogen evolves in a clock-like manner and accrues sufficient genetic diversity, the branching points in a phylogeny are likely to be very close to transmission events. A related complication is that the “transmission bottleneck” is not typically known; hosts may initially be infected with more than one pathogen lineage. Finally, hosts may be re-infected and carry multiple lineages as a consequence. We have chosen not to add these additional complexities to our exploration of memory and tree shapes. Indeed, if under models that capture within-host diversity or other complex features, transmission trees can be inferred^8^,^67^, then comparing the shapes of those transmission trees to what might be expected under different intensity functions, as presented here, remains relevant. Challenges in the next generation of phylodynamics have recently been discussed elsewhere^10^.

We have not focused on how sampling affects tree shapes, but the question of how sampling affects phylodynamic inference is a challenging one^10^. The density, timing and uniformity of sampling can be expected to affect shapes; in the limit of very low sampling density, we would expect the effects of non-exponential waiting times between infection events to be washed out by the fact that lineages in the sampled branching tree would change hosts many times between branching events (unless the low sampling is highly non-uniform). If sampling occurs through contact tracing, snowball sampling or respondent-driven sampling then this could have substantial effects on tree shapes, which are as yet uncharacterised.

In outbreak settings, many factors can impact tree shape, including *R*_0_, *M*, non-exponential distributions of waiting times (which we have explored here), but also including selection, population structure, host behaviour, super-spreading, host contact network structure^21^,^44^,^45^,^47^,^68^ and other factors. The complexity of the underlying models and the computational challenges associated with large datasets make likelihood-free inference an appealing tool in this domain^69^,^70^,^71^. However, this approach requires informative summary features that can be compared to properties of sequence data or to trees inferred from these data.

Tree shapes are potentially an important source of such summary features. The number of possible tree shapes explodes exponentially in the number of tips, so specifying a shape in principle specifies a lot of information. Using shapes as informative summary features will require much more finely-resolved shape statistics than the few that are currently in use – mainly imbalance and the number of cherries. We have found that tree shapes are quite robust to variations in the waiting times between the onset of infection and infecting others. However, in our study, some shapes were strongly constrained by data (tree height, the average length of connected “ladder” components and the imbalance) and some were strongly affected by high *R*_0_ and *M* (cherries, pitchforks, stairs and ILnumber). If this robustness to memory together with sensitivity to other aspects of the data, carries forward to an informative suite of tree shapes, shapes could provide an alternative approach to estimating epidemiological parameters such as *R*_0_ and *M* using sequence data.

## Additional Information

**How to cite this article**: Plazzotta, G. *et al.* Effects of memory on the shapes of simple outbreak trees. *Sci. Rep.* **6**, 21159; doi: 10.1038/srep21159 (2016).

## Supplementary Material

Supplementary Information

## Figures and Tables

**Table 1:** Details of the parameters that defined the simulations.

Scenario	Case	Intensity function	Life span	Final time	*R*_0_	*M*
Scenario 1	#1	*n* = 1/3, *k* = 1.50	no death	9.00	1.50	0.50
	#2	*n* = 2/3, *k* = 1.5	no death	9.00	1.50	0.35
	#3	*n* = 1, *k* = 1.5	no death	9.00	1.50	0.27
	#4	*n* = 4/3, *k* = 1.5	no death	9.00	1.50	0.22
	#5	*n* = 1/3, *k* = 2.21	no death	4.20	2.21	1.00
	#6	*n* = 2/3, *k* = 3.08	no death	4.20	3.06	1.00
	#7	*n* = 1, *k* = 4.30	no death	4.20	4.30	1.00
	#8	*n* = 4/3, *k* = 6.00	no death	4.20	6.00	1.00
Scenario 2	#1	*n* = 1/3, *k* = 1.50	no death	3.79	1.50	0.50
	#2	*n* = 2/3, *k* = 1.50	no death	5.54	1.50	0.35
	#3	*n* = 1, *k* = 1.50	no death	7.15	1.50	0.27
	#4	*n* = 4/3, *k* = 1.50	no death	8.93	1.50	0.22
	#5	*n* = 1/3, *k* = 2.21	no death	2.70	2.21	1.00
	#6	*n* = 2/3, *k* = 3.08	no death	3.20	3.06	1.00
	#7	*n* = 1, *k* = 4.30	no death	3.60	4.30	1.00
	#8	*n* = 4/3, *k* = 6.00	no death	3.70	6.00	1.00
Scenario 2-large	#1	*n* = 1/3, *k* = 1.50	no death	6.6	1.50	0.50
	#2	*n* = 2/3, *k* = 1.50	no death	9.3	1.50	0.35
	#3	*n* = 1, *k* = 1.50	no death	11.6	1.50	0.27
	#4	*n* = 4/3, *k* = 1.50	no death	14.6	1.50	0.22
	#5	*n* = 1/3, *k* = 2.21	no death	4.1	2.21	1.00
	#6	*n* = 2/3, *k* = 3.08	no death	4.7	3.06	1.00
	#7	*n* = 1, *k* = 4.30	no death	4.9	4.30	1.00
	#8	*n* = 4/3, *k* = 6.00	no death	5.2	6.00	1.00
Scenario 3	#1	*n* = 0, *k* = 2.31	exp, *δ* = 1.00	4.50	1.46	1.00
	#2	*n* = 1, *k* = 17.09	exp, *δ* = 1.00	4.50	1.46	3.97
	#3	*n* = 1, *k* = 126.29	exp, *δ* = 1.00	4.50	1.46	10.80
Scenario BD	NA	*k* = 1.5 ∀ *t*	exp, *δ* = 1.00	3.00	1.50	0.50
Scenario BDfit	NA	*k* = 7.38 ⋅ 10^−3^ ∀ t	exp, *δ* = 4.20 ⋅ 10^−3^	184	1.76	3.2 ⋅ 10^−3^

The choice of parameters was empirical, intended to generate trees with different intensity functions, i.e. effect of memory, sharing some biologically relevant measures such as *R*_0_, *M* or the size.

**Table 2:** Relation between shape topologies and memory, as included in our simulations.

	Shape topology	Behaviour		p-value	
		Cases 1–4	Cases 5–8	Cases 1–4	Cases 5–8
Scenario 1	Tips	decrease	decrease	<10^−15^	<10^−15^
	Colless	increase	increase	<10^−15^	1.4 ⋅ 10^−9^
	Sackin	increase	increase	<10^−15^	1.8 ⋅ 10^−14^
	Cherries	inconclusive	decrease	0.48	<10^−15^
	Pitchforks	increase	decrease	1.3 ⋅ 10^−6^	1.5 ⋅ 10^−4^
	Stairs2	increase	decrease	1.4 ⋅ 10^−4^	<10^−15^
	ILnumber	increase	increase	1.9 ⋅ 10^−5^	<10^−15^
	MaxHeight	increase	increase	<10^−15^	<10^−15^
	avgLadder	increase	increase	9.9 ⋅ 10^−9^	<10^−15^
Scenario 2	Colless	inconclusive	inconclusive	0.28	0.03
	Sackin	inconclusive	inconclusive	0.71	0.48
	Cherries	decrease	decrease	2.6 ⋅ 10^−4^	<10^−15^
	Pitchforks	inconclusive	decrease	0.9	5.4 ⋅ 10^−4^
	Stairs2	decrease	decrease	6.1 ⋅ 10^4^	<10^−15^
	ILnumber	increase	increase	3.9 ⋅ 10^−3^	<10^−15^
	MaxHeight	inconclusive	increase	0.47	3.9 ⋅ 10^−3^
	avgLadder	inconclusive	increase	0.23	1.1 ⋅ 10^−4^
Scenario 2-large	Colless	inconclusive	decrease	0.77	3.5 ⋅ 10^−4^
	Sackin	inconclusive	decrease	0.46	6.0 ⋅ 10^−5^
	Cherries	decrease	decrease	2.3 ⋅ 10^−4^	<10^−15^
	Pitchforks	increase	inconclusive	7.7 ⋅ 10^−4^	0.05
	Stairs2	decrease	decrease	8.0 ⋅ 10^−3^	<10^−15^
	ILnumber	increase	increase	6.1 ⋅ 10^−3^	<10^−15^
	MaxHeight	inconclusive	decrease	0.70	3.7 ⋅ 10^−3^
	avgLadder	inconclusive	inconclusive	0.82	0.29

Memory is introduced by means of step-like intensity functions which vary by step position and height; refer to Table 1 for the details of each simulation. For both groups (cases 1–4 and cases 5–8) and for each shape topology, a straight line was fitted to the data and here it is reported whether the slope was negative, positive or if the linear fitting was inconclusive because of a p-value greater than 0.01. Similar p-values were also obtained with an ANOVA test. Specific values for intercept and slope are not reported, because of the high specificity of parameters used in the simulations and the standardization of the shape topologies.
