# Supplementary material for: Effects of memory on the shapes of simple outbreak trees
Source: Sci Rep. 2016 Feb 18;6:21159. doi: 10.1038/srep21159 (PMC4758066; doi:10.1038/srep21159)
Supplement: Supplementary Information [file srep21159-s1.pdf]

Supplementary material of the article: Effects of memory on the shapes of simple outbreak trees

Giacomo Plazzotta[1], Christopher Kwan[2], Michael Boyd[3] and Caroline Colijn[1]

[1]Department of Mathematics, Imperial College London, London, UK
[2]Department of Electrical and Electronic Engineering, Imperial College London, London, UK
[3]Department of Mathematics, University of Cambridge, Cambridge, UK

October 20, 2015

The role of the distribution of secondary infections

It has been suggested [44, 46] that high variation in the number of secondary cases (the offspring distribution, in the terminology of branching processes), affects the shapes of branching trees. In the constant-rate birth death process, the offspring distribution is geometric. But in a non-homogeneous Poisson process the distribution is Poisson, at least when individuals survive their infectious period, as here. Accordingly, it is not possible to directly compare a memory-less process with a non-geometric offspring distribution to an analogous process with memory. Incorporating non-geometric offspring distributions into memory-less processes might be possible, for example using multi-type constant-rate processes, but it would not be straightforward. We simulated our inhomogeneous processes using a geometric offspring distribution with the same mean (instead of a Poisson), and illustrate the results in Figure S1. We find that the imbalance is somewhat higher, the stair feature is somewhat lower, and the average ladder components are somewhat more variable. However, overall these differences are minor; larger effects of the offspring distribution on shapes presumably require a more highly skewed distribution than we have used here.

The timing of branching events

In the main text, we focused on tree shapes because the shapes of real trees have long been known to be different than the shapes predicted by the Yule and continuous-time birth-death models [38, 42, 47], and recent work found that processes with memory provide a better match to data [47]. However, analyses of the timing of branching events in the tree are typically used in phylodynamic analysis [14, 13]. One simple approach to analysing the branch lengths for ultrametric trees is known as the γ statistic [62]; under the Yule model γ is expected to be 0. When $\gamma < 0$, branching events are further from the tips and when $\gamma > 0$ they are closer to the tips of the tree than would be expected under the constant rate assumption. As in [47], we analyse the branch lengths using γ; results are shown in Figure S2 for Scenarios 1 and 2 from the main text, and for the geometric offspring distibution. The values are negative in most cases. The length of time over which the simulations were run (to control the size

of the trees) had a pronounced effect in Case 8 (which has the highest R_0, the most shifted intensity function and a Malthusian parameter of 1; see Table 1. Here, the geometric offspring distribution had a quite pronounced effect, lowering the γ values significantly in Cases 1, 2, 3 and 8. Furthermore in cases 1-4 (where R_0 is the same and the Malthusian parameter M differs), M did not effect γ in the Poisson case but did in the geometric case. These results together with the birth rate fits presented in the main text suggest that analyses based on branch lengths are sensitive to memory in the underlying process, via both the waiting times (as would be expected intuitively) and the offspring distribution.

Correlations between tree shapes

We explored how the shape features are related to each other in the groups of trees. Figures S3-S6 illustrate correlations between shape features. We used the Spearman correlation and visualised it in matrix form using `corrplot` in the corrplot package in R. As described in the main text, shape correlations were remarkably robust to most of the variations we explored. The data-derived trees showed some differences, as did the more extremely-varying R_0 simulations in Scenario 3 (see main text).

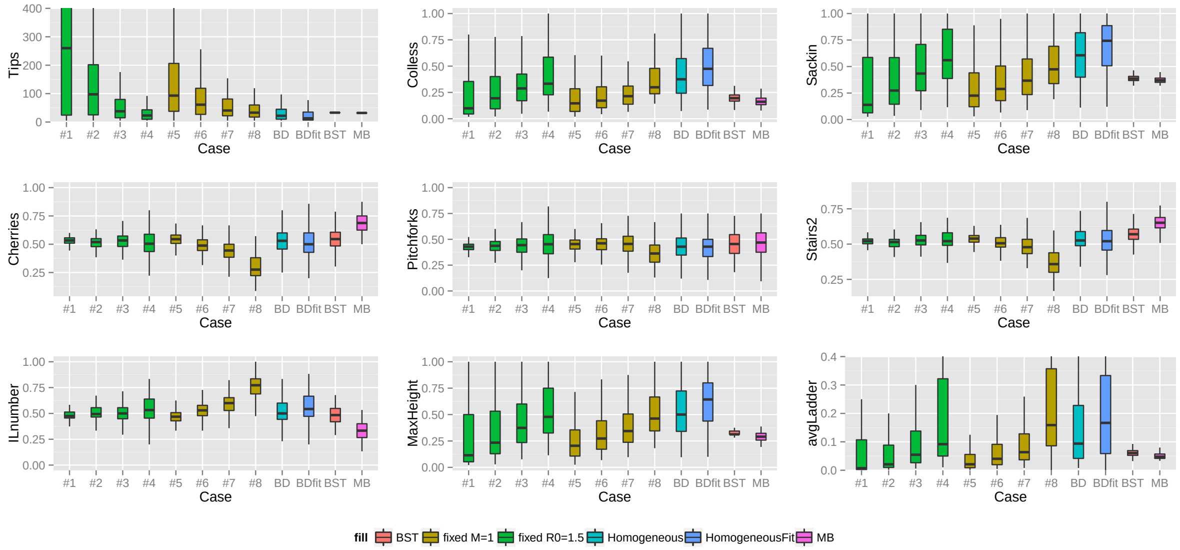

Figure S1: Tree shapes, as in Scenario 3, but with geometric offspring distribution rather than Poisson. Cases 1-4 have the same R_0 (1.5) and cases 5-8 have the same Malthusian parameter (1). BD refers to the constant-rate birth-death process with $R_0 = 1.5$, and BDFIT refers to the constant-rate birth-death process with parameters inferred from data. BST and MB are trees inferred from data using BEAST and MB respectively (see Table 1). BD, BDFIT, BST and MB trees are the same as in the figures in the main text, as the BD and BDFIT already had a geometric offspring distribution and the BST and MB trees are not affected by simulated offspring distributions.

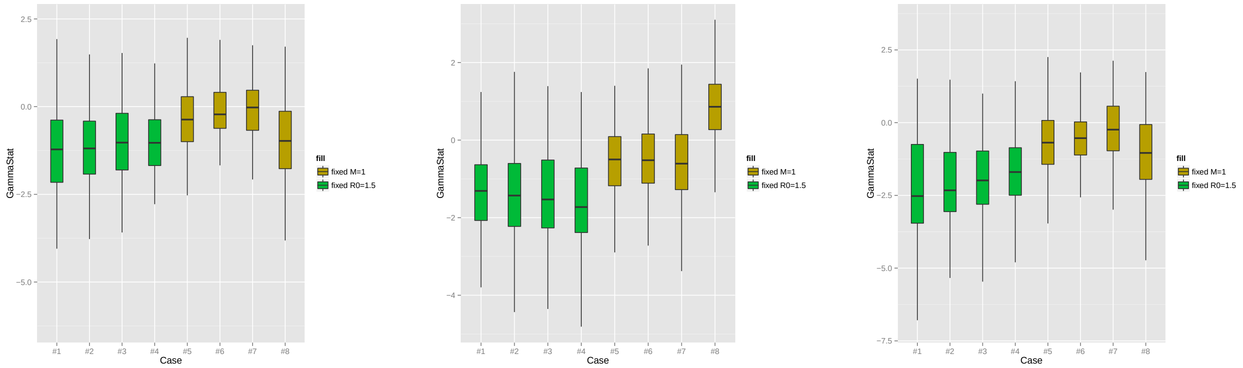

(a) Scenario 1 (size not controlled)

(b) Scenario 2 (size controlled)

(c) Geometric offspring (Sc. 1)

Figure S2: Pybus γ statistic for simulated trees

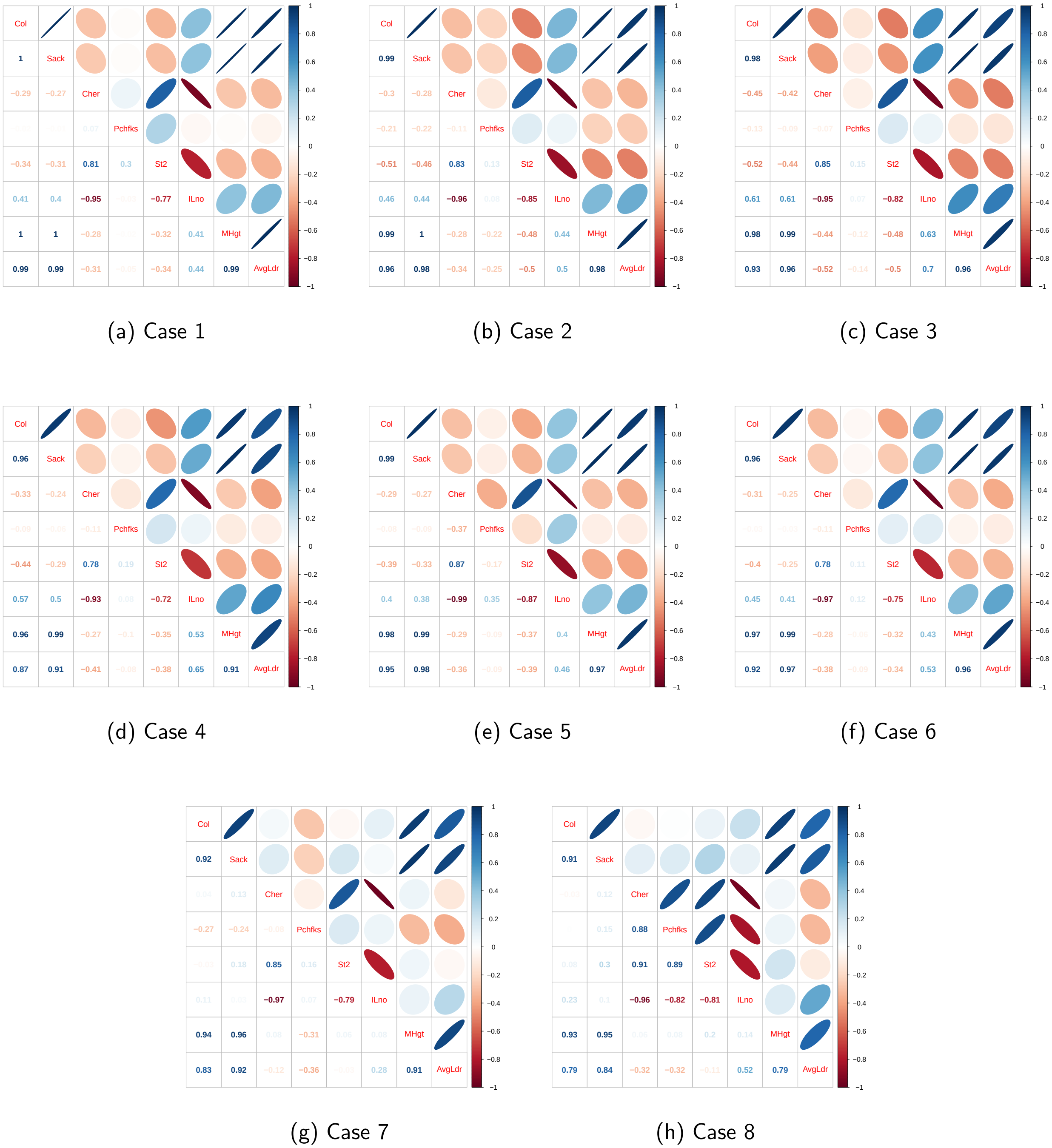

(a) Case 1 (b) Case 2 (c) Case 3

(d) Case 4 (e) Case 5 (f) Case 6

(g) Case 7 (h) Case 8

Figure S3: Correlations between shape features from Scenario 1. The colour indicates whether the correlation is positive (blue) or negative (red), and the slant of the blob in the upper panels indicates this as well. The colours are darker when the correlation is more significant. The Spearman correlation coefficients are shown on the lower diagonals; again darker text shows a more significant correlation.

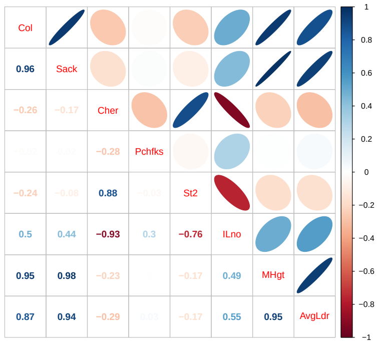

(a) Case 1

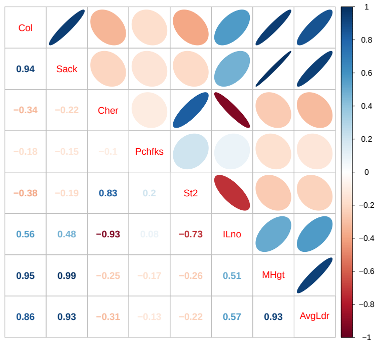

(b) Case 2

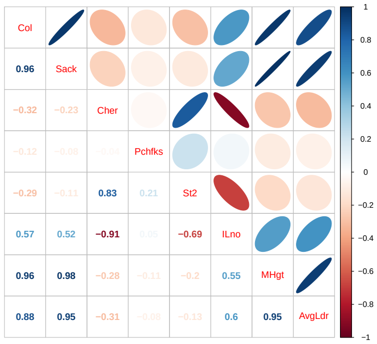

(c) Case 3

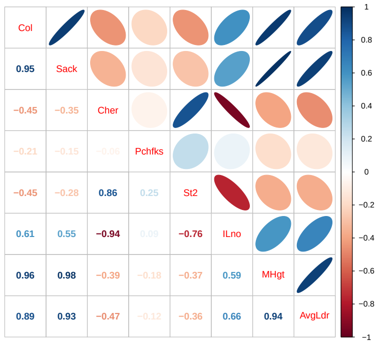

(d) Case 4

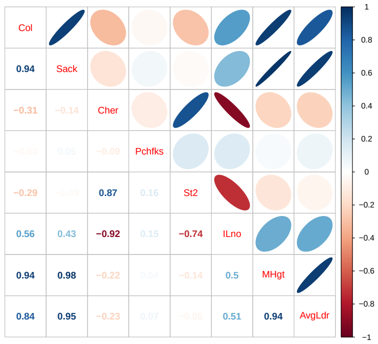

(e) Case 5

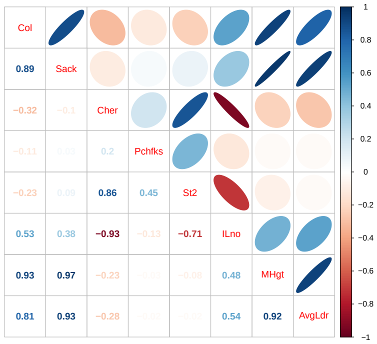

(f) Case 6

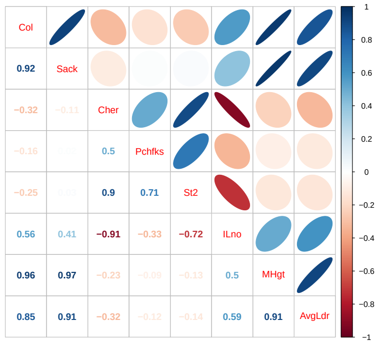

(g) Case 7

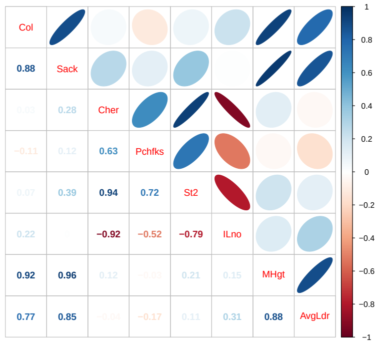

(h) Case 8

Figure S4: Correlations between shape features from Scenario 2. The colour indicates whether the correlation is positive (blue) or negative (red), and the slant of the blob in the upper panels indicates this as well. The colours are darker when the correlation is more significant. The Spearman correlation coefficients are shown on the lower diagonals; again darker text shows a more significant correlation.

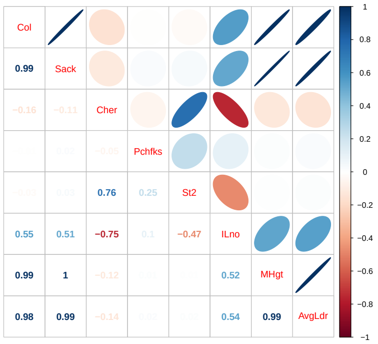

(a) Case 1

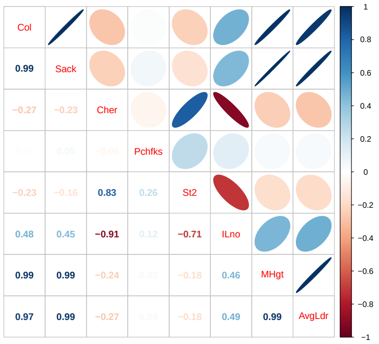

(b) Case 2

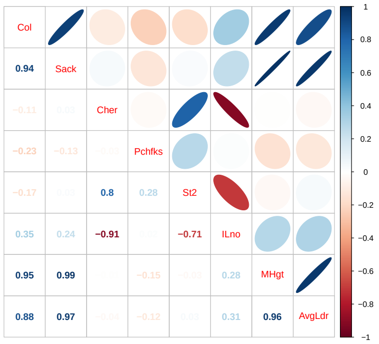

(c) Case 3

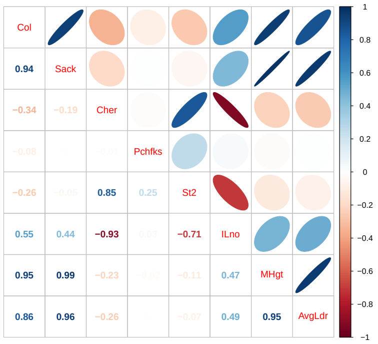

(d) Case 4

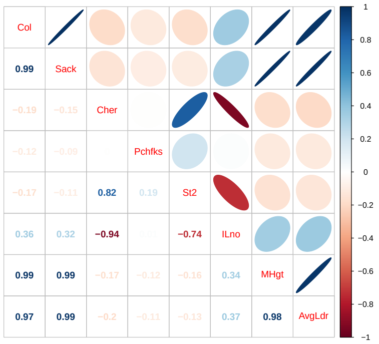

(e) Case 5

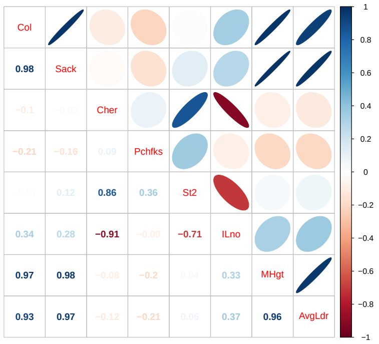

(f) Case 6

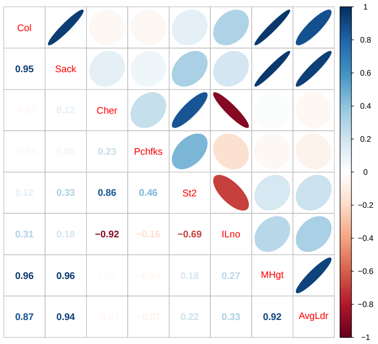

(g) Case 7

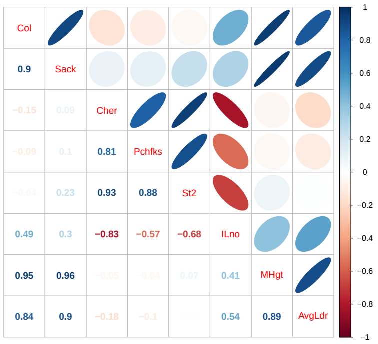

(h) Case 8

Figure S5: Correlations between shape features. The trees correspond to Figure S1 where the geometric offspring distribution was enforced. The colour indicates whether the correlation is positive (blue) or negative (red), and the slant of the blob in the upper panels indicates this as well. The colours are darker when the correlation is more significant. The Spearman correlation coefficients are shown on the lower diagonals; again darker text shows a more significant correlation.

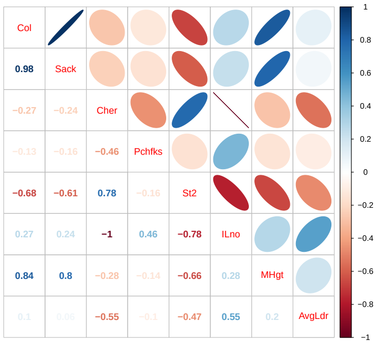

(a) Data: MrBayes

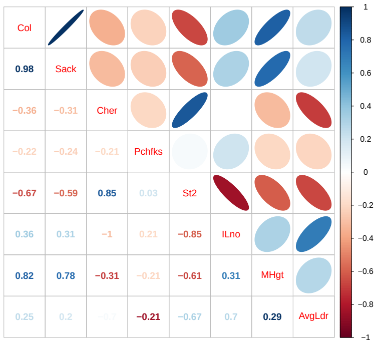

(b) Data: BEAST

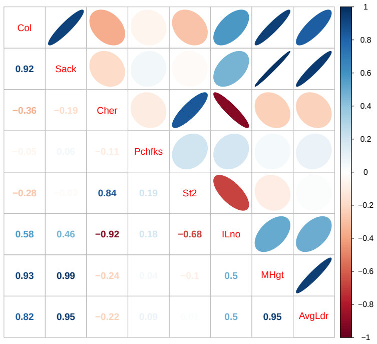

(c) Birth-death

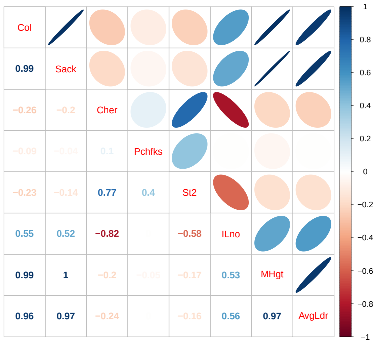

(d) R_0 1

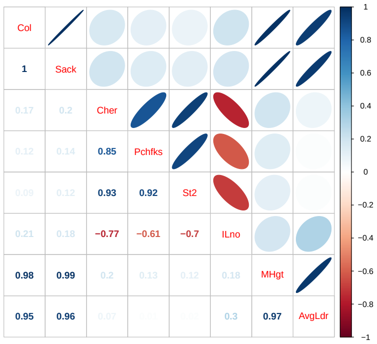

(e) R_0 2

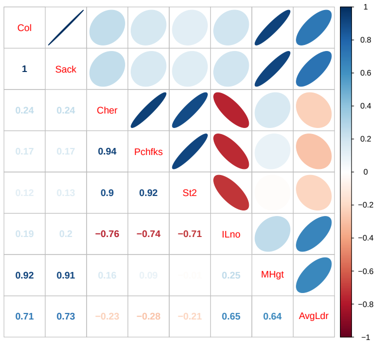

(f) R_0 3

Figure S6: Correlations between shape features for the data, the homogeneous trees, and the cases with widely varying R_0 (Scenario 3). The colour indicates whether the correlation is positive (blue) or negative (red), and the slant of the blob in the upper panels indicates this as well. The colours are darker when the correlation is more significant. The Spearman correlation coefficients are shown on the lower diagonals; again darker text shows a more significant correlation.
